# Descriptive account of the first use of the LeVe CPAP System, a new frugal CPAP System, in adult patients with COVID-19 Pneumonitis in Uganda

**DOI:** 10.1186/s41182-023-00533-9

**Published:** 2023-08-07

**Authors:** Anna Littlejohns, Helen Please, Racheal Musasizi, Stuart Murdoch, Gorret Nampiina, Ian Waters, William Davis Birch, Gregory de Boer, Nikil Kapur, Tumwesigye Ambrozi, Ninsiima Carol, Nakigudde Noel, Jiten Parmar, Peter Culmer, Tom Lawton, Edith Namulema

**Affiliations:** 1 Leeds Teaching Hospitals NHS Trust, Beckett Street, Leeds, LS9 7TF West Yorkshire UK; 2 Mengo Hospital, Sir Albert Cook Road, Mengo, P. O. Box 7161, Kampala, Uganda; 3 University of Leeds, Woodhouse Lane, Leeds, LS2 9JT UK; 4 Bradford Teaching Hospitals NHS Foundation Trust, Duckworth Ln, Bradford, BD9 6RJ UK

**Keywords:** LMIC, CPAP, NIV, COVID-19, Pandemic, Sustainability, Anaesthetics, Global Health

## Abstract

**Background:**

Continuous positive airway pressure (CPAP) has been a key treatment modality for Coronavirus Disease 2019 (COVID-19) worldwide. Globally, the demand for CPAP outstripped the supply during the pandemic. The LeVe CPAP System was developed to provide respiratory support for treatment of COVID-19 and tailored for use in low- and middle-income country (LMIC) settings. Prior to formal trial approval, received in November 2021, these devices were used *in extremis* to support critically unwell adult patients requiring non-invasive ventilatory support.

**Methods:**

This is a retrospective descriptive review of adult patients with COVID-19 pneumonitis, who were treated with advanced respiratory support (CPAP and/or high-flow nasal oxygen, HFNO) at Mengo Hospital, Uganda. Patients were treated with the LeVe CPAP System, Elisa CPAP and/or AIRVO™ HFNO. Treatment was escalated per standard local protocols for respiratory failure, and CPAP was the maximum respiratory support available. Data were collected on patient characteristics, length of time of treatment, clinical outcome, and any adverse events.

**Results:**

Overall 333 patients were identified as COVID-19 positive, 44 received CPAP ± HFNO of which 43 were included in the study. The median age was 58 years (range 28–91 years) and 58% were female. The median duration of advanced respiratory support was 7 days (range 1–18 days). Overall (all device) mortality was 49% and this was similar between those started on the LeVe CPAP System and those started non-LeVe CPAP System devices (50% vs 47%).

**Conclusions:**

The LeVe CPAP system was the most used CPAP device during the pandemic, bringing the hospital’s number of available HFNO/CPAP devices from two to 14. They were a critical resource for providing respiratory support to the sickest group of patients when no alternative devices were available. The devices appear to be safe and well-tolerated with no serious adverse events recorded. This study is unable to assess the efficacy of the LeVe CPAP System; therefore, formal comparative studies are required to inform further use.

## Introduction

### Coronavirus Disease 2019 (COVID-19) pandemic in Uganda

Africa was one of the later continents to be hit by COVID-19. Following the catastrophic consequences of the pandemic in Asia and Europe, there was huge concern regarding the potential damage COVID-19 could have on already fragile health systems [1–4]. There was also concern over potential higher mortality due to complications related to the geographic prevalence of certain comorbidities, particularly the high human immunodeficiency virus burden [5, 6]. The first case of COVID-19 in Uganda was detected on 21st March 2020; and the World Health Organisation has since reported 170,369 confirmed cases of COVID-19 and 3630 deaths from COVID-19 in Uganda to date [7, 8]. These cases occurred in 3 main ‘waves’: August 2020–January 2021, May–October 2021, and December 2021–January 2022 [7]. By far the highest number of cases and mortalities occurred in the second wave, with 2873 deaths. This wave made up 79% of total COVID-19 deaths to date in Uganda [8].

Initially, the guidance for management of severe COVID-19 respiratory failure favoured early intubation [5]. However, later studies demonstrated the utility of early continuous positive airway pressure (CPAP), with good safety and efficacy; as well as reducing the number of patients progressing to require intubation and mechanical ventilation, avoiding the added risks of invasive mechanical ventilation [6, 9–11]. Similarly, studies showed the importance of high-flow nasal oxygen (HFNO) as an alternative non-invasive technique to manage acute hypoxemic respiratory failure in COVID-19 patients outside the intensive care setting [12]. HFNO has been shown to be superior to conventional oxygen therapy, is well-tolerated and reduces the need for invasive ventilation [12–16].

In Uganda, as with many low- and middle-income countries (LMICs), there are huge limitations for patients to receive high dependency or intensive care, invasive or non-invasive ventilation (NIV) [1, 17–21]. These limitations stem from a multitude of well-known factors including aspects of infrastructure, human resources, local expertise, equipment availability and maintenance, training levels of healthcare staff, and financial burden to patients and relatives [17]. In Uganda, adult intensive care medicine has been under-prioritised, leaving it unprepared for a surge in demand, with a limited capacity of only 55 functional critical care beds for a population of around 40 million [17, 19, 21]. Therefore, tools for managing patients outside of critical care are crucial in this setting, hence the prioritisation of building CPAP capacity locally.

### Frugal CPAP device

**Fig. 1:**
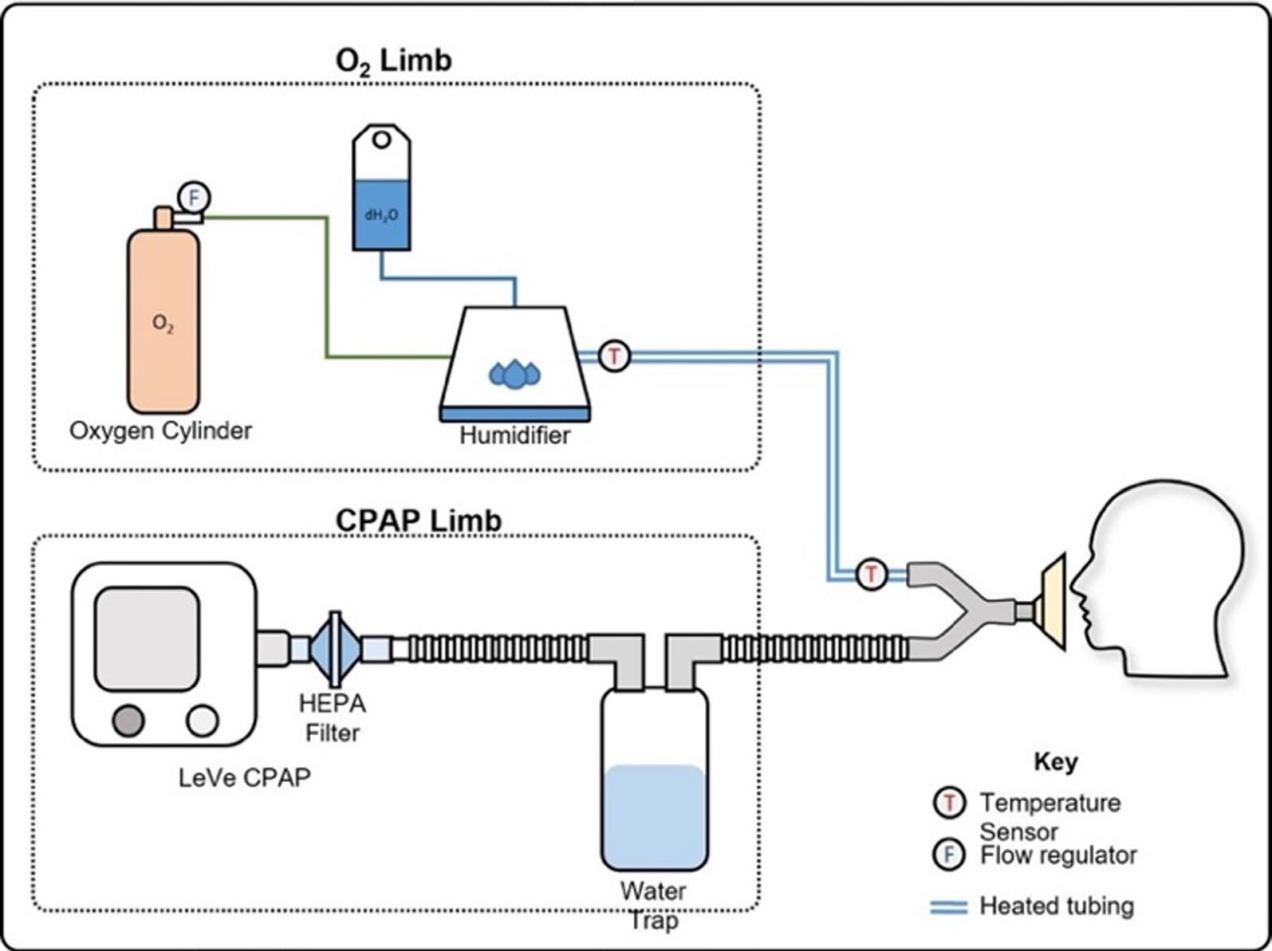
LeVe CPAP System using the 'Racheal' breathing circuit: featuring the LeVe CPAP oxygen delivery device coupled with a customised breathing circuit designed by Ugandan biomedical engineers to meet local resource and clinical care needs at Mengo Hospital, Uganda

The LeVe CPAP device is a novel CPAP device designed through collaboration between Mengo Hospital and The University of Leeds, in response to the COVID-19 pandemic [22]. It has been developed using frugal engineering techniques to tailor the technology to LMIC settings. The LeVe CPAP device uses an electric fan to supply the necessary airflow, which inherently has the appropriate flow dynamics required to safely deliver the required CPAP pressures. It can deliver a range of pressures through varying the fan speed. A four-way dial allows selection of different pressure settings (5, 7.5, 10, 12.5 cmH_2_O) [22]. The device generates pressurised air flow independent of the supplied oxygen. The device has a simple expiration port and does not require a positive end expiratory pressure (PEEP) valve thereby reducing complexity and cost. The fraction of inspired oxygen (FiO2) is controlled by the oxygen flow rate and thus is controlled independently of CPAP pressures. Oxygen efficiency is key to making the devices affordable and sustainable, and in the context of COVID-19 preserving precious oxygen supplies was even more critical [23]. Biomedical engineers at Mengo Hospital developed a breathing circuit for the LeVe CPAP device to meet their available resource and clinical needs. The resultant LeVe CPAP System (comprising the LeVe CPAP device and the ‘Rachael’ breathing circuit) is shown in Fig. 1.

With the overwhelming demand for respiratory support during the COVID-19 pandemic [9] Mengo Hospital and The University of Leeds worked together to design a formal study examining the use of LeVe CPAP System in COVID-19 patients. Twelve LeVe CPAP devices were provided to Mengo Hospital for this purpose. The first stage of clinical testing was a pilot study in healthy adults at Mengo Hospital. The results were encouraging, demonstrating the devices caused no harm in terms of oxygen saturation levels or end tidal carbon dioxide levels, as well as being well-tolerated by users [22]. The next stage was planned to be a crossover trial using the LeVe CPAP System and other commercially available HFNO and CPAP devices available at Mengo Hospital (AIRVO™ and Elisa). However, prior to receiving formal ethical approval the unprecedented and overwhelming clinical demand for advanced respiratory support devices became so great that the hospital supported use of the available LeVe CPAP Systems ahead of the trial.

This paper gives a descriptive account of the use of the LeVe CPAP System in this extreme situation, where NIV availability was severely limited, and demand was outstripping supply considerably. The aim of the study is to examine the use of the LeVe CPAP System amongst critically unwell adults with COVID-19 pneumonitis at Mengo Hospital, Uganda.

## Methods

This is a retrospective observational descriptive study. We identified all patients admitted to Mengo Hospital with a positive COVID-19 polymerase chain reaction (PCR) who were treated with advanced respiratory support (HFNO and/or CPAP) at any point during their admission. HNFO was delivered using an AIRVO™ device which was procured by the hospital in response to the pandemic. CPAP was delivered using two different types of devices: the new LeVe CPAP System, and the Elisa ventilator using its non-invasive mode. The Elisa ventilators were used in the Mengo Hospital critical care unit prior to the pandemic for both invasive and non-invasive ventilation. During the pandemic one of these was moved to the COVID-19 isolation wards to allow NIV delivery. We collected data on all patients escalated to HFNO/CPAP and recorded which device (or devices) the patients were treated with. Patients were escalated to advanced respiratory support by the clinical team as per the usual local hospital protocols for hypoxaemic respiratory failure. CPAP was the highest level of respiratory support on the COVID-19 isolation ward at Mengo Hospital. At the time of the clinical decision to escalate to advanced respiratory support, the patients were commenced on one of the devices purely based on availability. All cases were treated between 1st May 2021 and 30th September 2021 inclusive.

### Data collection

Data was collected through review of the hospital’s hand-written case notes. The data collected included patient characteristics, relevant past medical history, device used, length of time on advanced respiratory support, clinical outcome at end of admission (i.e., discharge on room air, discharge on home oxygen, onward referral, death) and any complications.

### Statistical analysis

Owing to the retrospective nature of this study, the relatively low numbers in each group, and the amount of crossover with patients using multiple devices, we have focussed on providing descriptive data to give an insight into the pandemic response as opposed to statistical analysis.

### Choice of study location

The study location was Mengo Hospital based on Kampala, Uganda. Mengo Hospital is a relatively well-resourced East African hospital which functions as a private, not-for-profit hospital, requiring patients to pay for their care. It was the first hospital established in East Africa in 1897 and has just celebrated its 125-year anniversary. It has many departments including medicine, surgery, obstetrics, gynaecology, anaesthetics, intensive care, paediatrics, neonatology, HIV counselling, psychiatry, a resuscitation room, and outpatient departments. Mengo Hospital also has onsite laboratory, pharmacy services, and a blood bank. As with many hospitals globally, Mengo had to redesign their ward structure during the pandemic. All adult COVID-19 patients were exclusively treated on repurposed COVID-19 isolation wards.

Mengo Hospital was deemed an appropriate site for using these devices. First, the clinical and engineering teams at Mengo Hospital had been integral collaborators both academically and clinically in the creation and initial testing of the LeVe CPAP System and staff were familiar with device use. Second, the hospital has anaesthesiologists experienced in providing CPAP to adults. Third, the hospital was accredited by the Ugandan Ministry of Health to treat COVID-19 patients. Finally, Mengo Hospital has a history of research expertise.

### Ethics

The use of the LeVe CPAP System in COVID-19 was started prior to receiving formal ethical approval for their planned trial. This was deemed justified due to the overwhelming need for respiratory support of critical cases of respiratory failure. Given the lack of alternative ventilatory support locally, it was deemed appropriate to make use of these devices clinically in a number of the most unwell patients.

Given the device was used prior to ethical approval and outside of a trial context, our team felt there was an ethical obligation to collect and share the data regarding its use and outcomes. We deemed that a retrospective study of routine case notes would not require formal ethical approval. We obtained administrative approval and guidance from the local institutional review board. The project had local support from the hospital management team and the treating teams.

The pilot study involving healthy volunteers at Mengo Hospital had approval from Mengo Hospital Research and Ethics Committee, The Uganda National Council for Science and Technology and The Pan African Clinical Trials Registry [22].

## Results

**Fig. 2:**
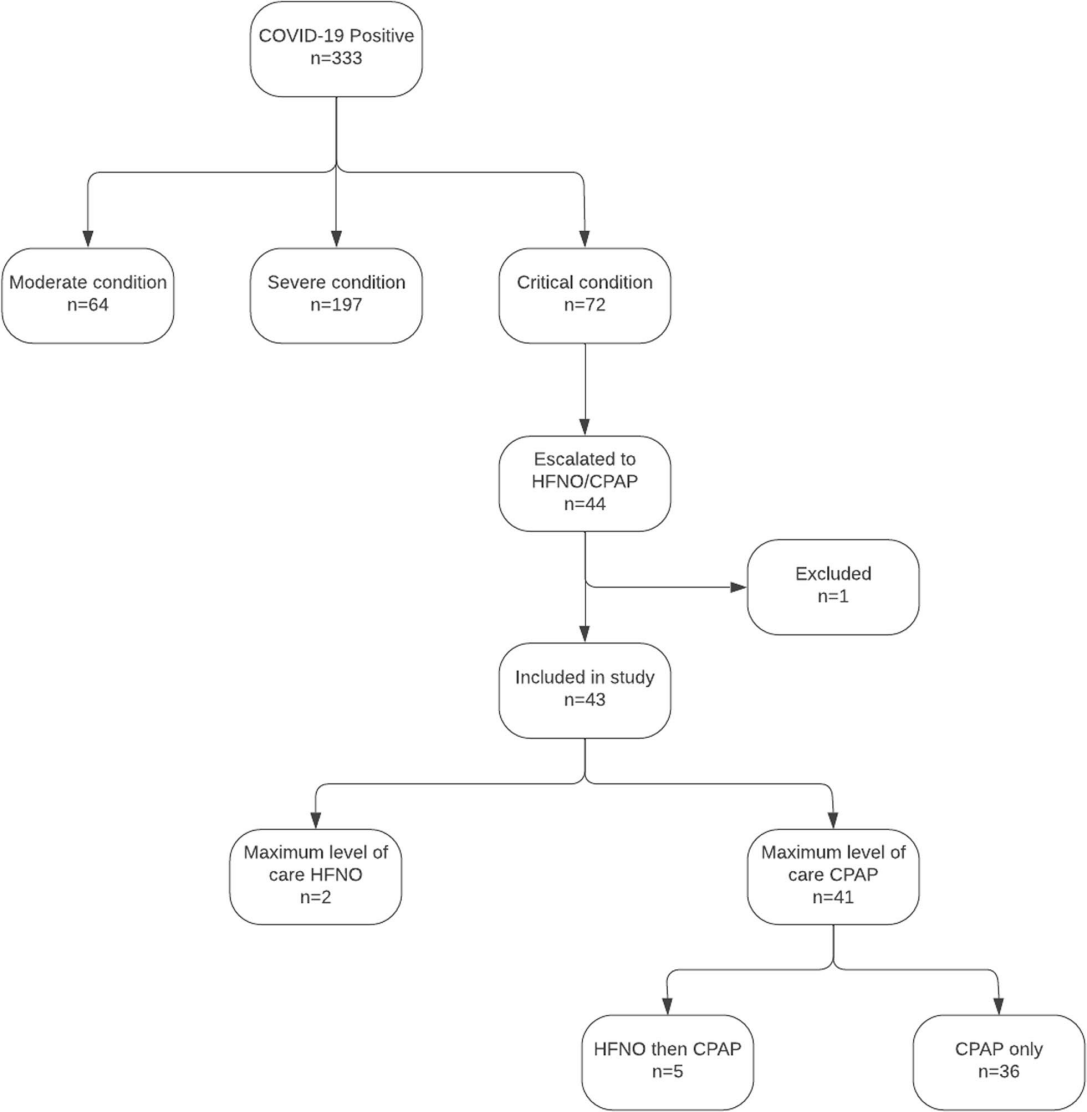
Numbers of patients admitted to Mengo Hospital in 2021 with positive COVID-19 PCR results who were escalated to advanced respiratory support and included in the study

Overall, 333 inpatients were identified as COVID-19 positive on PCR testing at Mengo Hospital. In total 277 of these patients were discharged home, while 56 died in hospital. More admissions were females than male (205 vs 128), with 64 of the 205 females either pregnant or recently post-partum. Most of the patients were Ugandan (311) with a low number of non-nationals and refugees (22). Clinically, 72 were deemed to have critical disease, as demonstrated in Fig. 2, and 44 patients were escalated to advanced respiratory support. All these cases were treated between 1st May 2021 and 30th September 2021 inclusive.

**Fig. 3:**
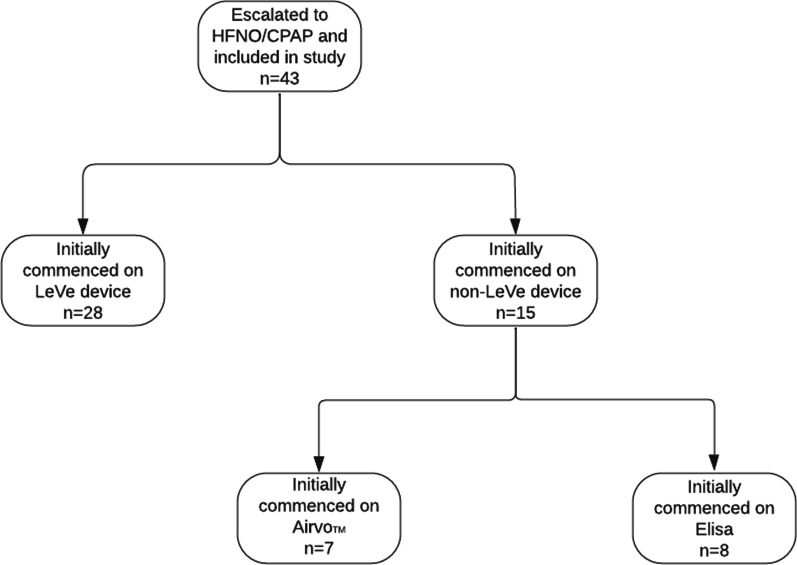
Different devices patients were commenced on when escalated to advanced respiratory support

As shown in Fig. 2, the number of patients initially identified as eligible for our review was 44 with the inclusion criteria of adult inpatient, COVID-19 positive PCR and received advanced respiratory support. One patient was excluded, as no case notes could be located, leaving a total of 43 patients being included in our review. Figure 2 also shows the breakdown of the level of support patients received (HFNO, CPAP or a combination). Figure 3 shows which device patients were commenced on when initially escalated to HFNO/CPAP.

Table 1 demonstrates the comorbidities recorded, with hypertension and diabetes mellitus being the most common. Patients managed with advanced respiratory support had a median age of 58 years (range 28–91 years) and 58% were female, further breakdown of characteristics is provided in Tables 2 and 3. A number of patients were commenced on one device but subsequently received treatment with another device. To reflect this the data are displayed in several ways. Table 2 compares patients treated with a single device and patients treated with a combination of devices, and Table 3 compares those treated totally or initially with LeVe CPAP System, and those treated totally or initially by AIRVO™ or Elisa (non-LeVe CPAP System). Table 3 demonstrates that baseline patient characteristics are largely similar between those different device groups. The rate of hypertension was higher in the non-LeVe CPAP System group (39% vs 47%) and a larger difference was seen in the rate of diabetes mellitus (18% vs 40%).

**Table 1:** Distribution of documented comorbidities in the 43 patients receiving CPAP/HFNO

Comorbidities		Number of patients
Number of comorbidities in total group (Total = 43 patients)	0	13
	1	8
	2	7
	3	5
	4	1
	Unknown/not documented	9
Frequency of comorbidities (In the 21 patients with documented comorbidities)	Hypertension	18
	Diabetes Mellitus	11
	Obesity	3
	Asthma	3
	Concurrent infection (excluding HIV)	3
	HIV	2
	Heart Failure	1

**Table 2:** Demographics and outcomes of patients who received HFNO/CPAP, categorised by those who were treated with a single device and those who were treated with a combination of devices

		Treatment with single device	Treatment with combination of devices
Total	Number	32	11
Sex	Male	11 (34%)	7 (64%)
	Female	21 (66%)	4 (36%)
Age (years)	Range	28–91	41–77
	Median	58	55
Duration on CPAP (days)	Range	1–12	4–18
	Mean	6	9
	Median	6	9
Outcome at hospital discharge	Died	13 (41%)	8 (73%)
	Discharged	17 (53%)	2 (18%)
	Referred	2 (6%)	1 (9%)

**Table 3:** Demographics and outcomes of patients who received HFNO/CPAP, categorised by those who were initially treated with the LeVe CPAP Systems and those who were treated with a non-LeVe CPAP Systems

		Initial treatment with LeVe CPAP System	Initial treatment with non-LeVe CPAP System
Total	Number	28	15
Sex	Male	11 (39%)	7 (47%)
	Female	17 (61%)	8 (53%)
Age	Range	28–91	45–77
	Median	55	57
Duration on CPAP	Range	1–18	1–9
	Mean	7	6
	Median	8	5
Outcome	Died	14 (50%)	7 (47%)
	Discharged	11 (40%)	8 (53%)
	Referred	3 (10%)	0 (0%)

The duration of time on HFNO/CPAP ranged from 1 to 18 days, with a median of 7 days. Duration of treatment was longer in those initially or totally treated with LeVe CPAP System compared to those treated totally or initially by non-LeVe CPAP System (median 8 days vs 5 days, respectively). Overall mortality was 49% (21/43). The mortality for those who were treated using a single device was 40% (13/32) and for those treated with a combination of device types was 73% (8/11). The mortality of those initially or totally treated with LeVe CPAP System was 50% and for those treated totally or initially by non-LeVe CPAP System was 47%. Regarding complications during admission, one patient developed a pulmonary embolism, two developed sepsis, and two developed diabetic ketoacidosis. Two patients had been referred to Mengo Hospital from other healthcare centres, and three patients were referred onwards to other hospitals from Mengo. The indication for onward referrals was identified as financial cost to patient/family in every case. One patient was discharged with home oxygen. 

## Discussion

From this retrospective study we are able to describe the safe use of the newly implemented LeVe CPAP System, and comment on the patient demographics and overall mortality.

The second wave of the pandemic by far was the most difficult in Uganda with the highest incidence of disease and highest mortality [7, 8]. By this stage, global experience and literature had demonstrated the importance of CPAP as a tool to manage moderate to severe cases, and CPAP had been shown to reduce the need for invasive mechanical ventilation [9, 17, 18, 24]. This was key as ventilator availability and critical care capacity was stretched globally, with particular concern in Uganda and other LMICs with limited critical care capacity [1, 19]. The availability of CPAP in Uganda and other LMICs, is unfortunately severely limited, even outside of the pandemic [25]. There is very little literature on use of CPAP in adults in Uganda to gauge its level and distribution of use, but there are a number of barriers to its implementation. These barriers include: the expense of the initial device, the expense of maintenance, the availability of suitable and reliable oxygen and electricity sources, the availability of appropriate monitoring (e.g., saturation probes) and the need for appropriate training for healthcare staff. The LeVe CPAP System was designed specifically for LMIC settings, making use of frugal engineering to overcome these challenges, as outlined in the introduction.

Our results demonstrate the importance of the LeVe CPAP System at Mengo Hospital, in the context of COVID-19-related respiratory failure. CPAP was the highest level of respiratory support available in our setting. The LeVe device was the most used CPAP device providing non-invasive ventilatory support to patients who would not have been able to receive that otherwise. They hugely increased the availability of functioning devices; there were 12 LeVe devices, compared to only one Elisa ventilator and one AIRVO™ device for HFNO; resulting in a 700% increase in the hospital’s total number of available HFNO/CPAP delivery devices. Without the LeVe CPAP Systems there would have been a critical shortage of devices and only a fraction of these critically ill patients would have received this treatment. These patients had already failed management on conventional oxygen therapy, so without being able to escalate to advanced respiratory support they would likely have had much worse outcomes. Owing to collaborative efforts, the LeVe CPAP Systems were able to be implemented during the peak of the pandemic. It is encouraging to see that not only were the devices physically available but there was enthusiasm from the local clinicians who were competent and confident with their use.

On reviewing case notes there were no significant adverse effects identified related to the LeVe CPAP Systems or any of the CPAP/HFNO devices. Potential unwanted effects from CPAP use could have been pressure-related damage to face, excess leakage from mask or intolerance of mask. One patient using the LeVe CPAP System found the nose and mouth mask uncomfortable which led to the team subsequently ordering full face masks as an alternative for future use. The overall mortality in the CPAP/HFNO group was 49%. This appears comparable with published studies of COVID-19 outcomes from high dependency and intensive care settings in Africa [26].

Eleven patients were treated sequentially with different devices. This reasoning in each case was hard to fully comprehend from a retrospective review of the case notes but appeared to be for a variety of reasons. When a patient was requiring escalation beyond conventional oxygen therapy, they commenced whichever device was available at that time. Largely it appears that if patients were deteriorating on HFNO they would then receive CPAP when a device was available. If patients were already receiving treatment with CPAP (via Elisa or LeVe CPAP Systems) and continued to deteriorate sometimes clinicians chose to change device in case the alternative proved to be superior in that case. This worked both ways between Elisa and LeVe CPAP Systems. It should be remembered that these were the sickest COVID-19 patients the hospital managed, all deemed as ‘critical’. This change of device is an indicator of the clinicians trying any strategy possible with the resources available. A prospective trial comparing these devices had not been undertaken at the time of the devices being used, so superiority of one device was not known, and it appears reasonable that clinicians adopted this approach. The data demonstrate better outcomes for patients who were treated on one device compared to those who were treated with a combination of devices sequentially. This is as expected, given the reasons articulated above that it was those who were initially critical and then continued to deteriorate that were moved between devices. 

There was a low onward referral rate, with only three patients in the cohort being referred to another facility, all for financial reasons. This was expected, as Mengo Hospital is a private not-for-profit hospital, where patients must pay for their care, and it is usual practice that patients who are struggling financially to meet healthcare costs at Mengo Hospital may be referred onward to government hospitals. The low referral rate may, therefore, indicate acceptability of the cost of CPAP at Mengo Hospital. The LeVe CPAP System oxygen consumption is lower than alternative devices, which in turn reduces cost to patients; which may be a factor in acceptability.

## Limitations

This was a small single centre retrospective review. It was not designed to be large enough for meaningful statistical analysis or inter-group comparisons. A lot of patients were treated with multiple devices, again making comparisons between groups challenging. There was also some limitation of documentation in relation to device type in several cases. This meant there was a need for corroboration of notes by cross-referencing with the key COVID-19 response staff who were able to review the notes and confidently confirm device type based on documentation review. The unintended benefit of this was that those reviewing the case notes were in fact blinded to device type. This study only included critically ill patients; therefore, we lack data on outcomes of using CPAP/HFNO in moderately ill patients, in the context of potentially preventing further deterioration. Unfortunately, we lack information on the pre-hospital functional level of these patients. This was not routinely recorded in the case notes, so we have not been able to provide this and have only been able to provide information on comorbidities. 

## Conclusions

These preliminary results show that CPAP therapy was crucial to the care of patients with COVID-19 pneumonitis in Mengo Hospital during the second wave of the pandemic. The LeVe CPAP Systems were a critical resource when faced with unprecedented demand for ventilatory support in critically unwell patients. In this extreme situation our results demonstrate that the LeVe CPAP Systems were employed safely and successfully and were able to provide NIV to several patients who would have had no alternative NIV support.

This has been a truly collaborative piece of work, comprising both interdisciplinary collaboration between engineering, academic and clinical teams, as well as intercontinental collaboration between teams based in Leeds (UK), Bradford (UK) and Kampala (Uganda).

It is difficult to comment on the efficacy of the device or compare groups statistically based on the relatively low numbers and retrospective nature of this study. However, the results do indicate there is sufficient promise to the LeVe CPAP System to prompt further investigation through more formal comparative studies. We also hope to see the LeVe CPAP System being taken forwards to different clinical scenarios aside from COVID-19.

## Data Availability

Data are available on reasonable request.

## Abbreviations

COVID-19: Coronavirus Disease 2019
CPAP: Continuous positive airway pressure
HFNO: High-flow nasal oxygen
LMIC: Low- and middle-income country
NIV: Non-invasive ventilation
Fi02: Fraction of inspired oxygen
PCR: Polymerase chain reaction
PEEP: Positive end expiratory pressure
